# A Chart Review on the Feasibility and Safety of the Vincristine Irinotecan Pazopanib (VIPaz) Association in Children and Adolescents With Resistant or Relapsed Sarcomas

**DOI:** 10.3389/fonc.2020.01228

**Published:** 2020-08-06

**Authors:** Ida Russo, Virginia Di Paolo, Alessandro Crocoli, Angela Mastronuzzi, Annalisa Serra, Pier Luigi Di Paolo, Angela Di Giannatale, Evelina Miele, Giuseppe Maria Milano

**Affiliations:** ^1^Department of Pediatric Hematology/Oncology, Bambino Gesù Children's Hospital, IRCCS, Rome, Italy; ^2^Department of Surgery - Bambino Gesù Children's Hospital, IRCCS, Rome, Italy; ^3^Department of Radiology, Bambino Gesù Children's Hospital, IRCCS, Rome, Italy

**Keywords:** vincristine, irinotecan, pazopanib, pediatric sarcomas, new drugs

## Abstract

**Background:** Pediatric patients with relapsed or refractory sarcomas have poor outcome and need novel therapies that provide disease control while maintaining an acceptable quality of life. The safety of vincristine, irinotecan, and pazopanib (VIPaz) association has not yet been published in this population.

**Methods:** A chart review was conducted in children and adolescents with relapsed or refractory bone and soft tissue sarcomas who received VIPaz in our institution.

**Results:** One hundred sixty-six patients with a diagnosis of soft or bone sarcoma were admitted to our hospital in the period between March 2015 and August 2018, 30 were relapsed or resistant. Seventeen out of 30 resistant or relapsed patients (median age, 14 years) received 114 VIPaz cycles (median six cycles per patient, range 1–17). Sixteen courses (15%) resulted in gastrointestinal toxicity with Grade two diarrhea; 35 courses (30%) resulted in Grade ≥3 neutropenia. One patient presented Grade two hypothyroidism after nine courses, and another one had Grade two hyperbilirubinemia after 12 courses. Two and five patients required a 25% dose reduction of irinotecan (because of diarrhea) and pazopanib (because of neutropenia four and hyperbilirubinemia 1), respectively. No patient experienced heart failure, hypertension, nor posterior reversible encephalopathy syndrome. Pneumothorax was not reported in any case even in lung metastatic patients. After two and four VIPaz cycles, we observed one complete response (CR), five partial responses (PRs), seven stable diseases (SDs), and four progressive diseases (PDs). With a median follow-up of 15 months (range 3–32), five out of 17 (29%) patients were alive, and four patients were in continuous CR after 12 VIPaz cycles.

**Conclusions:** The VIPaz regimen might be a safe option in children and adolescents with relapsed or refractory sarcomas otherwise unable to be enrolled in other clinical trials; on the other hand, the efficacy of pazopanib observed cannot be sustained from the current study.

## Introduction

Sarcomas represent 10% of cancers in children and 8% in adolescents and young adults (1). Except for a few aggressive histotypes, the outcome has greatly improved due to the use of intensive chemotherapy combination, staging refinement, and more effective local control by surgery and irradiation. On the other hand, patients with relapsed or refractory tumors have an extremely poor outcome, with only 10% of patients alive at 5 years from diagnosis (2, 3). Thus, a continuous effort is underway to identify new effective treatments in this patients' setting. In the last two decades, irinotecan, a camptothecin analog, has been largely used as salvage therapy for relapsed or refractory sarcomas. Taking into consideration the two most frequent histotypes, rhabdomyosarcoma (RMS) and Ewing sarcoma (EWS), two irinotecan-containing regimens—associated with vincristine (VI) and temozolomide (IT), respectively—have been reported to improve survival in relapsed or refractory patients (4, 5). Many other international experiences have been published after these early reports, with several schedules associating different antineoplastic drugs with irinotecan (6–11), but without superior results to those with VI or IT. Many trials worldwide considered VI association as the backbone of choice for new drug combinations, being a regimen well-known and well-tolerated.

We decided to combine VI with a new anti-vascular endothelial growth factor (VEGF) molecule, pazopanib (VIPaz), given its promising results achieved in adult sarcomas (12, 13). Pazopanib is an oral pan-tyrosine kinase inhibitor (TKi) targeting VEGF receptor (VEGFR-1,−2,−3), c-Kit, and platelet-derived growth factor receptor (PDGFR) (14). In the largest phase III trial conducted for adult sarcomas (13), pazopanib reduced the risk of progression or death compared to placebo; main reported toxicities were fatigue, hypertension, anorexia, and diarrhea. Sporadically, thromboembolic events and left ventricular ejection fraction drop were reported in the pazopanib arm. This drug is nowadays approved in Italy for the treatment of relapsed sarcoma and advanced renal cell carcinoma in adulthood (15). Despite the promising results of pazopanib in adult soft tissue sarcomas, few experiences of its use in the pediatric setting were published. A phase I study involving 51 children affected by refractory solid tumors identified the maximum tolerated dose (MTD) for both formulations, tablets (450 mg/m^2^/day) and suspension (160 mg/m^2^/day) (16). Among evaluable patients, results reported were two partial responses (PRs) [one desmoplastic small round cell tumor (DSRCT) and one hepatoblastoma) and eight stable diseases (SDs) (seven out of with sarcoma) (16). To date, no phase II studies have been disclosed yet. Here we describe our experience on 17 patients with relapsed or refractory sarcomas who received VIPaz regimen as off-label compassionate use. To our knowledge, this is the first report of pazopanib combination in a pediatric and adolescent population.

## Patients and Methods

A chart review of patients affected by sarcoma admitted between March 2015 and August 2018 at the Bambino Gesù Children Hospital, one of the main pediatric oncology centers in Italy, was done. All patients had histologically proven soft or bone sarcoma. For all patients, the histological diagnosis was revised by an experienced pathologist. Patients treated with VIPaz were identified using pharmacy administration records. An institutional review board approved the study, finalized to export data from hospital records; data thus selected are demographics, histology, radiology information, treatment administration, inpatient admissions, and status at data analysis. Data sources include hospital records, histopathologic registries (bone and soft tissue malignancies keywords), radiologic and laboratory records, pharmacy records (pazopanib keywords).

The presented data were censored at December 1, 2019.

### Therapy

The treatment schedule was as follows: irinotecan 50 mg/m^2^/day intravenously for 5 days; vincristine 1.5 mg/m^2^ intravenously, administrated on days 1 and 8 of each cycle; and pazopanib 450 mg/m^2^ once daily (maximum 600 mg/day) *per os* per 21 days of each cycle. Pazopanib was given with clear liquids at least 1 h before or 2 h after a meal. Cefixime, 8 mg/kg once daily (maximum 400 mg/day), was administered orally, during the first week of each cycle to prevent irinotecan-associated diarrhea. If diarrhea occurred, loperamide was given and microbiological investigations were performed. The whole therapy was given on an outpatient basis. Informed consent was obtained from parents or legal guardians.

### Assessment of Toxicity and Response

Toxicities were determined and graded using the Common Terminology Criteria for Adverse Events version 4.0 (2009).

Measurable and evaluable disease, as well as disease response (both primary tumor and metastases, if present), was defined and assessed according to the National Cancer Institute (NCI) Response Evaluation Criteria in Solid Tumors (RECIST version 1.1). The objective response rate (ORR) [complete response (CR) + PR] and the tumor control rate (TCR) (CR + PR + SD) were reported after two VIPaz courses with a binomial exact confidence interval as the percentage of participants who have a CR, PR, or SD as determined by investigator assessment of response in accordance with RECIST version 1.1 (17). Overall survival (OS) and time to progression (TTP) were calculated according to the Kaplan–Meier method.

## Results

One hundred sixty-six patients with a diagnosis of soft or bone sarcoma were admitted to our hospital in the period between March 2015 and August 2018, of which 25 relapsed and five were resistant to the first-line treatment. Seventeen patients out of 30 were treated at our department with the VIPaz regimen as second-line or third-line therapy.

Patients and tumors characteristics and final outcome are listed in Table 1.

**Table 1:** Patient's clinical characteristics.

**Characteristics**	**Value**
**Gender**	
Female	9
Male	8
**Histology**	
ARMS	5
ERMS	3
EWS	5
CIC-DUX	1
US	1
CCS	1
DSRCT	1
**Mts at initial diagnosis**	
Lung	4
Bone	3
BM	2
**Peritoneal carcinomatosis**	1
None	9
**Response after 2 cycles**	
CR	1
PR	5
PD	4
SD	7
**Age at diagnosis**	
≥1 <10	3
<1 ≥10	14
**Primary site**	
Thorax	2
Extremity	8
HN PM	3
Axial	1
Pelvis	2
Unknown	1
**Disease status**	
Relapse	10
Refractory tumor	1
PD on 1st line therapy	5
PD on 2st line therapy	1
**Outcome**	
DOD	12
NED	4
AWD	1

*ARMS, alveolar rhabdomyosarcoma; ERMS, embryonal rhabdomyosarcoma; EWS, Ewing Sarcoma; CIC-DUX, CIC-DUX fusion transcript positive sarcoma; US, undifferentiated sarcoma; CCS, clear cell sarcoma; DSRCT, desmoplastic round cell tumor; HN PM, Head and neck parameningeal; Mts, metastasis; BM, bone-marrow; VIP, Vincristine/Irinotecan/Pazopanib; PD, progressive disease; CR, complete response; PR, partial response; SD, stable disease; DOD: died of disease; NED, no evidence of disease; AWD, alive with disease*.

No patient had a previous treatment with other inhibitors of angiogenesis or VEGF or similar drugs. None had previous irinotecan treatments.

The median age was 14 years (range 5–19 years). At the time of treatment start, one patient had a refractory tumor, five patients had progressive disease (PD) (two local; three had combined local and metastatic tumor), and 10 patients had relapsing tumors (three local, seven metastatic). Diagnoses included RMS (*n* = 8; five alveolar and three embryonal), EWS (*n* = 5), clear cell sarcoma (*n* = 1), CIC Fusion with Double-Homeobox (DUX) Transcription Factors (CIC-DUX) fusion-transcript-positive sarcoma (*n* = 1), undifferentiated sarcoma (*n* = 1), and DSRCT (*n* = 1). Previous treatment received by the patients included anthracycline and alkylating agent-based chemotherapy, radiotherapy (seven patients), and in one case, high-dose chemotherapy (busulfan-melphalan).

### Toxicity

One hundred fourteen cycles of VIPaz (median six cycles per patient; range 1–17) were extrapolated from pharmacy data source. Toxicities were reported in Table 2.

**Table 2:** Events occurred.

**No. of patients (%)**		
**Events**	**All grade**	**Grade ≥ 3**
Anorexia	8 (47%)	0
Diarrhea	7 (41%)	2^*^ (12%)
Fatigue	13 (76%)	0
Fever	0	1 (9%)
Hair hypopigmentation	0	0
Hypertension	0	0
Hypothiroidism	1 (9%)	0
Left ventricular systolic dysfunction	0	0
Liver disorder	1 (9%)	0
Nausea	10 (59%)	0
Neutropenia	8 (47%)	6° (35%)
Pneumothorax	0	0
Rash	1(9%)	0
Sinus bradycardia	0	0
Vomiting	4 (23%)	0

* *2 cases with Cl. Difficile infection °3 pts had Neutropenia G3 after the 9th course*.

Gastrointestinal toxicity with Grade 2 diarrhea occurred in 16 cycles (15%), and in two cases, *Clostridium difficile* was isolated. Grade ≥3 neutropenia occurred in 35 cycles (30%). One patient needed hospitalization and granulocyte colony-stimulating factor (G-CSF) support because of neutropenic fever with a negative blood culture. One patient with alveolar RMS presented Grade 2 hypothyroidism after the 9th VIPaz cycle and needed thyroid hormone supplements; he did not have prior head and neck radiotherapy nor high-dose chemotherapy. Another patient with DSRCT presented Grade two hyperbilirubinemia after the 12th VIPaz cycle not related to the disease. Two and five patients required a 25% dose reduction of irinotecan (because of diarrhea) and pazopanib (because of neutropenia four and hyperbilirubinemia 1), respectively. No patient experienced heart failure nor posterior reversible encephalopathy syndrome or pneumothorax.

### Response and Outcome

After two cycles of VIPaz regimen, we observed one CR, five PRs, seven SDs, and four PDs. The ORR was 47% (95% CI, 16–68%), and the TCR was 82% (95% CI, 38–88%). All these responses were confirmed after the fourth course. Local treatment was done after the fourth course of therapy. Only two patients had local treatment: in patient 5, radiotherapy was administered for residual lung metastasis, and in patient 12 after 10 courses, radiotherapy was done before surgery to the vertebra (see Supplementary Table 1).

**Figure 1:**
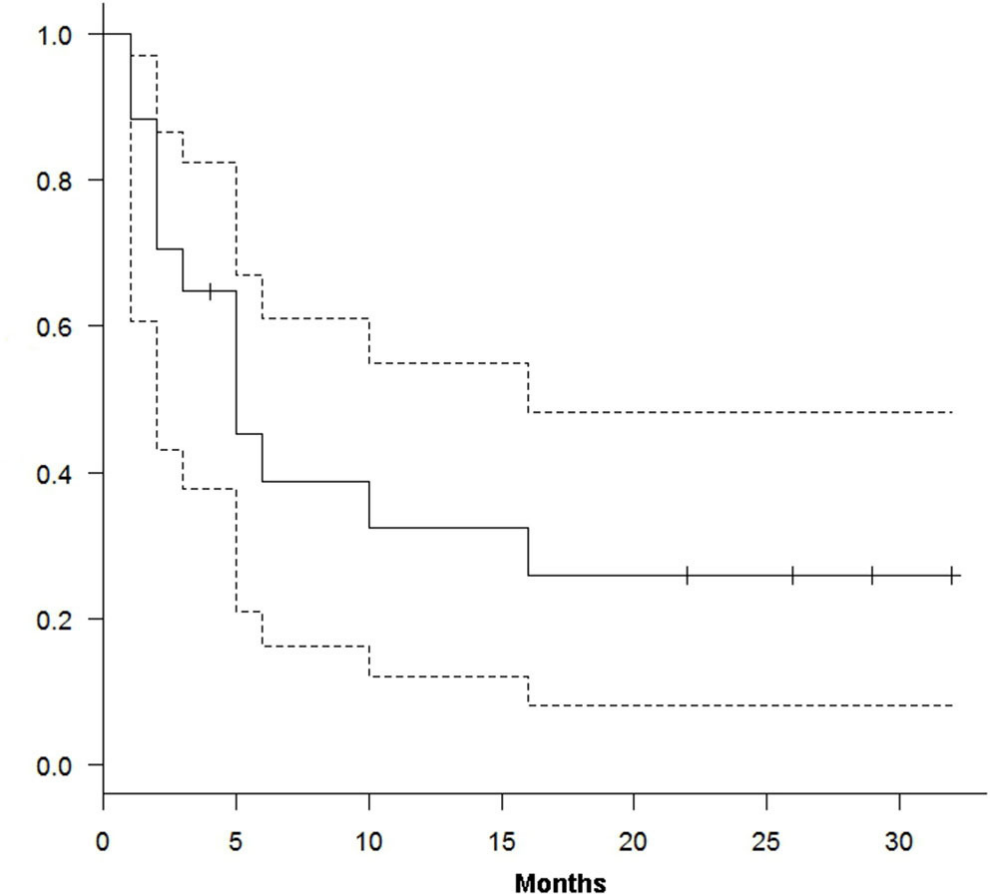
Kaplan–Meier curve for time to progression.

**Figure 2:**
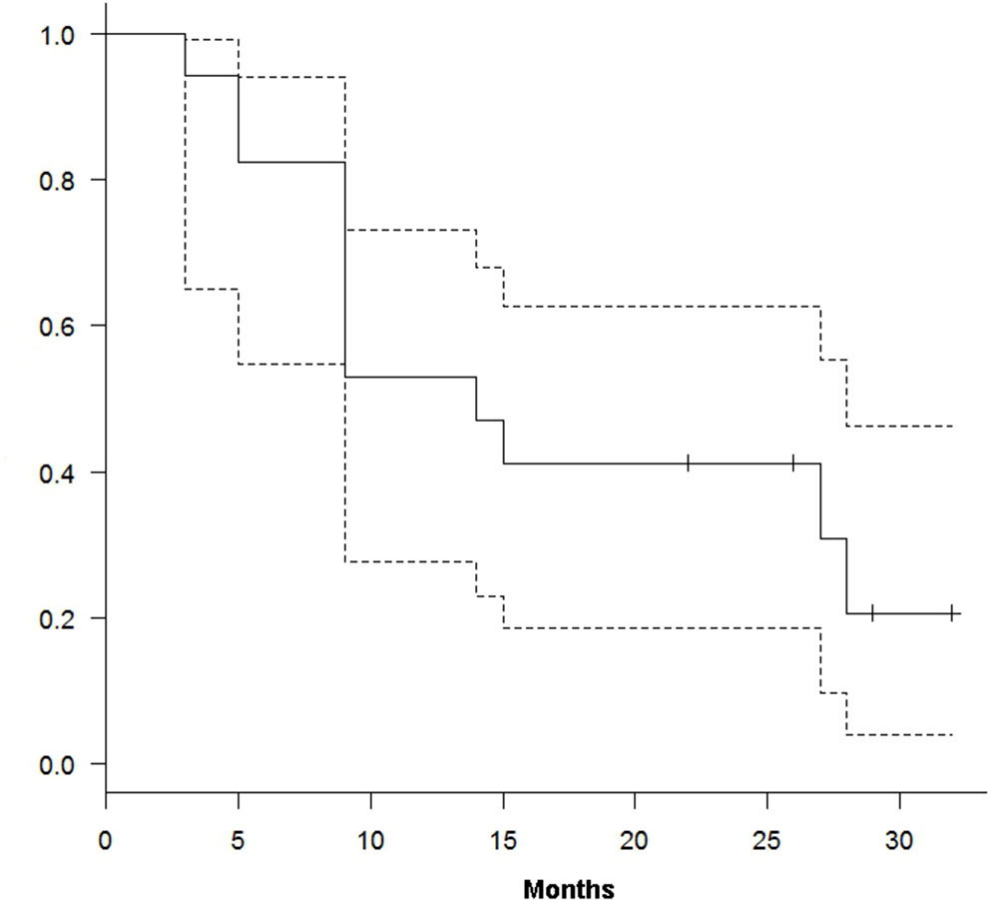
Kaplan–Meier curve for overall survival.

At a median follow-up of 15 months (range 2–32), 12 out of 17 patients have died of disease and five patients are alive (four in CR and one in PD). We obtained a median TTP and OS of 10 (range 1–32) and 15 (range 3–32) months, respectively. From the treatment start, TTP and OS were 39% (95% CI, 16–67%) and 82% (95% CI, 55–94%) at 6 months and 26% (95% CI, 11–81%) and 47% (95% CI, 23–68%) at 12 months (Figures 1, 2).

## Discussion

Although the survival of pediatric sarcomas has increased dramatically in the last decades, the prognosis for the refractory/relapsed cases is still dismal.

During the last two decades, several regimens have been adopted as second- and third-line therapies in this patient setting, where it is a strong need for new effective therapies that combine the classic mechanisms of action of chemo with new molecular mechanisms. The VI combination was the precursor of many salvage regimens projected to this scope. From this assumption, we designed VIPaz with the aim to offer a compassionate and safe therapeutic option for patients unable to be enrolled in other ongoing clinical trials.

This is the first report that describes that VIPaz is safe and well-tolerated in children and adolescents with refractory sarcoma.

Because of the small population reviewed, we cannot compare toxicities with an internal cohort of a controlled set of patients in the same period of time.

No severe adverse events or discontinued treatment was reported during treatment; only five patients needed 25% pazopanib dose reduction, and in two patients, a 25% irinotecan dose reduction.

None of our patients had hypertension or pneumothorax. We had a case of hyperbilirubinemia after 12 courses, probably related to the pazopanib, rapidly normalized after dose reduction. Hematological toxicity, mainly neutropenia, was observed with VIPaz. While, with respect to the data emerging from the literature on the association of IT or VI/VIT (4–19), we had similar gastrointestinal toxicities or less, we observed a slightly higher percentage of neutropenia (30%), especially if it compared with the 16% reported by (4) with VI alone.

On the other hand, early data of VIT-0910 trial (NCT01355445), where relapsed or resistant RMS patients were randomized to VIT/VI, showed hematological toxicity grade ≥3 significantly increased in the VIT arm (81 vs. 59%, *P* = 0.02) (20). Publication is not yet available to argue a possible comparison between the safety of VIT or VIPaz.

In this report, we observed one confirmed CR after four VIPaz cycles in a patient with relapsed alveolar RMS (patient 2, Supplementary Table 1); one persistent PR in a patient with embryonal head and neck RMS who relapsed in the lung and at the time of analysis still in CR after 12 VIPaz courses plus radiotherapy (patient 5, Supplementary Table 1); one patient with vertebral EWS (patient 12, Supplementary Table 1) who progressed during first-line therapy and treated with 10 VIPaz cycles followed by radiotherapy plus radical surgery. Another patient with undifferentiated chemoresistant sarcoma (patient 13, Supplementary Table 1) obtained a durable PR and long-term survival. Interestingly, we observed a long-lasting SD in a patient with DSRCT (patient 15, Supplementary Table 1).

Several clinical trials are ongoing worldwide in order to improve the poor prognosis of resistant sarcomas mainly using VIT regimen. Combining VI to temozolomide (VIT), Raciborska et al. (18) and Setty et al. (19) obtained a TCR (CR + PR + SD) of 68% in EWS and 26% in RMS patients, respectively. In the study by Raciborska et al. (18), the reported 1-year OS was 40%. Certainly, in our study population, we obtained a TCR of 82% and 1-year OS of 47% in a pool of different diseases, and the data shown, albeit anecdotal on RMS, might be better evaluated in a larger sample.

Although it is largely recognized that sarcomas express several pro-angiogenic therapeutic targets, only a few trials with anti-VEGF inhibitors have been published in pediatric sarcomas mainly using bevacizumab (21–24). In preclinical and clinical studies, it has been demonstrated that pazopanib has activity in sarcomas, also as a single-agent therapy. Moreover, in preclinical sarcoma models, pazopanib has shown additive or synergistic effects in combination with chemotherapies (25). Nowadays, pazopanib has been rarely included in pediatric studies. Indeed, there are few clinical trials investigating pazopanib in children: one phase II single-agent trial in relapsed/refractory solid tumors, conducted by COG (NCT01956669), results not yet available; one phase I, open-label, multicenter trial testing pazopanib in combination with irinotecan and temozolomide in children and young adults with relapsed or refractory sarcoma (NCT03139331) and still recruiting.

Lastly, pazopanib has also been included in the COG protocol ARST1321 as a neoadjuvant therapy in non-rhabdo soft tissue sarcoma that can be removed by surgery. Preliminary results of these trials are not yet available.

Despite our limits, due to the monocentric chart review of a small cohort of patients with sarcomas of heterogeneous subtypes, we might state that the VIPaz regimen might be a safe therapy in pediatric patients with refractory or relapsed sarcomas not otherwise treated with other regimens or included in phase I–II clinical trials. Of course, the efficacy of pazopanib cannot be ascertained from the current study.

Prospective trials are necessary to further explore such treatment options in a selected population.

## Data Availability Statement

All datasets generated for this study are included in the article/Supplementary Material.

## Ethics Statement

The studies involving human participants were reviewed and approved by Bambino Gesù Children's hospital ethics committee. Written informed consent to participate in this study was provided by the participants' legal guardian/next of kin.

## Author Contributions

GM drew the study and scheme, wrote the paper, and performed statistical analysis. EM, VD, and AD edited and revised the manuscript. IR, AS, AM, and AC managed the patients and revised manuscript. PD reviewed all radiological studies.

## Conflict of Interest

The authors declare that the research was conducted in the absence of any commercial or financial relationships that could be construed as a potential conflict of interest.

## Abbreviations

VIPaz: Vincristine/irinotecan/pazopanib
RMS: Rhabdomyosarcoma
EWS: Ewing sarcoma
VI: Irinotecan/vincristine
IT: Irinotecan/temozolomide
TKi: Tyrosine kinase inhibitor
VEGFR: Vascular endothelial growth factor receptor
PDGFR: Platelet-derived growth factor receptor
MTD: Maximum tolerated dose
DSRCT: Desmoplastic small round cell tumor
TC: Computed tomography
MRI: Magnetic resonance imaging
NCI: National Cancer Institute
RECIST: Response Evaluation Criteria in Solid Tumors
CR: Complete response
PR: Partial response
PD: Progressive disease
SD: Stable disease
ORR: Objective response rate
TCR: Tumor control rate
OS: Overall survival
TTP: Time to progression
VIT: Vincristine/irinotecan/temozolomide.
