# Supplementary material for: A Chart Review on the Feasibility and Safety of the Vincristine Irinotecan Pazopanib (VIPaz) Association in Children and Adolescents With Resistant or Relapsed Sarcomas
Source: Front Oncol. 2020 Aug 6;10:1228. doi: 10.3389/fonc.2020.01228 (PMC7425124; doi:10.3389/fonc.2020.01228)
Supplement: Supplementary file 1 [file Table_1.DOCX]

| Pt # | Age (Y)/  Gender | Histology | Primary site | Mts at initial diagnosis | Disease status | N° cycles  administered | Response after 2 cycles | FU (m)/  Outcome |
| --- | --- | --- | --- | --- | --- | --- | --- | --- |
| 1 | 15/M | ARMS | Thorax | None | Relapse | 9 | PR | 15/DOD |
| 2 | 17/F | ARMS | Thorax | Lung | Relapse | 13 | PR | 14/DOD |
| 3 | 16/M | ARMS | Unknown | Bone; BM | Relapse | 1 | PD | 2/DOD |
| 4 | 14/F | ARMS | Extremity | BM | Relapse | 2 | PD | 5/DOD |
| 5 | 5/M | ERMS | HN PM | None | Relapse | 12 | PR | 26/NED |
| 6 | 9/F | ERMS | HN PM | None | Refractory Tumor | 2 | SD | 32/NED |
| 7 | 10/M | ERMS | HN PM | None | Relapse | 2 | SD | 10/DOD |
| 8 | 17/M | CIC-DUX | Extremity | Lungs | PD on 1st line therapy | 3 | PD | 5/DOD |
| 9 | 14/F | EWS | Extremity | Bone | Relapse | 1 | PD | 3/DOD |
| 10 | 19/M | EWS | Extremity | None | PD on 1st line therapy | 7 | SD | 9/DOD |
| 11 | 15/F | EWS | Extremity | None | Relapse | 4 | PR | 9/DOD |
| 12 | 11/F | EWS | Axial | None | PD on 1st line therapy | 10 | SD | 29/NED |
| 13 | 11/M | US | Extremity | None | Relapse | 12 | PR | 27/DOD |
| 14 | 11/F | CCS | Pelvis | Lung; Bone | PD on 1st line therapy | 3 | SD | 9/DOD |
| 15 | 12/F | EWS | Extremity | Lung | PD on 2^nd^ line | 7 | SD | 15/AWD |
| 16 | 12/F | ARMS | Exstremity | None | Relapse | 12 | CR | 22/NED |
| 17 | 15/M | DSRCT | Pelvis | Peritoneal carcinomatosis | PD on 1st line therapy | 17 | SD | 28/DOD |

**SUPPLEMENTARY TABLE 1 Characteristics of the 17 patients treated with VIP regimen**

**Pt:** patient; **Y:** years; **M:** male; **F:** female; **Mts:** metastasis; **VIP:** Vincristine/Irinotecan/Pazopanib; **N°:** number; **FU:** follow-up; **m:** months; **ARMS:** alveolar rhabdomyosarcoma; **ERMS:** embryonal rhabdomyosarcoma; **CIC-DUX:** CIC-DUX fusion transcript positive sarcoma; **EWS:** Ewing Sarcoma; **US:** undifferentiated sarcoma; **CCS:** clear cell sarcoma; **DSRCT:** desmoplastic round cell tumor; **HN PM:** Head and neck parameningeal; **BM:** bone-marrow; **CR:** complete response; **PR:** partial response; **SD:** stable disease; **PD:** progressive disease; **NED:** no evidence of disease; **DOD:** died of disease; **AWD:** alive with disease; **NA:** not available
